# Static Plantar Pressure under Different Conditions in Children with Surgically Treated Unilateral Slipped Capital Femoral Epiphysis

**DOI:** 10.3390/children11040496

**Published:** 2024-04-20

**Authors:** Marius Negru, Andrei Daniel Bolovan, Elena Amaricai, Liliana Catan, Oana Belei, Adrian Emil Lazarescu, Corina Maria Stanciulescu, Eugen Sorin Boia, Calin Marius Popoiu

**Affiliations:** 1 Doctoral School, “Victor Babes” University of Medicine and Pharmacy, 300041 Timisoara, Romania; marius.negru@umft.ro; 2 Research Center for Assessment of Human Motion, Functionality and Disability, “Victor Babes” University of Medicine and Pharmacy, 300041 Timisoara, Romania; amaricai.elena@umft.ro (E.A.); catan.liliana@umft.ro (L.C.); 3 Department of Rehabilitation, Physical Medicine and Rheumatology, Faculty of Medicine, “Victor Babes” University of Medicine and Pharmacy, 300041 Timisoara, Romania; 4 First Pediatric Clinic, Disturbance of Growth and Development on Children Research Center, “Victor Babes” University of Medicine and Pharmacy, 300041 Timisoara, Romania; belei.oana@umft.ro; 5 Department of Anatomy, Faculty of Medicine, “Victor Babes” University of Medicine and Pharmacy, 300041 Timisoara, Romania; lazarescu.adrian@umft.ro; 6 2nd Clinic of Orthopaedics and Traumatology, Timisoara Emergency County Hospital, 300723 Timisoara, Romania; 7 Teodor Sora Research Centre, Department of Orthopaedics and Traumatology, “Victor Babes” University of Medicine and Pharmacy, 300041 Timisoara, Romania; 8 Department of Pediatric Surgery, “Victor Babes” University of Medicine and Pharmacy, 300041 Timisoara, Romania; stanciulescu.maria@umft.ro (C.M.S.); boia.eugen@umft.ro (E.S.B.); mcpopoiu@umft.ro (C.M.P.)

**Keywords:** slipped capital femoral epiphysis, assessment, foot load

## Abstract

Background: Slipped capital femoral epiphysis (SCFE) is the most common hip disease during infancy and adolescence. Our study aimed to analyze static plantar pressure in children with surgically treated unilateral SCFE. Methods: Twenty-two children with right SCFE with in situ fixation with one percutaneous screw were assessed by PoData plantar pressure analysis under three different conditions (open eyes, eyes closed, and head retroflexed). Results: The total foot loading was significantly higher on the unaffected limb compared with the affected one for all the three testing conditions (*p* < 0.05). When assessing the differences between testing conditions, there were no significant differences for the right and left foot loadings, or for the three sites of weight distribution, except for the right fifth metatarsal head (lower loading in eyes-closed condition in comparison to eyes open, *p* = 0.0068), left fifth metatarsal head (increased loading in head-retroflexed condition in comparison to eyes open, *p* = 0.0209), and left heel (lower loading in head-retroflexed condition in comparison to eyes open, *p* = 0.0293). Conclusion: Even after a successful surgical procedure, differences in foot loading can impact the postural static activities in different conditions (natural eyes-open, eyes-closed, or head-retroflexed posture).

## 1. Introduction

Slipped capital femoral epiphysis is a prevalent hip disease that occurs during infancy and adolescence. The incidence of this disease increases continuously, primarily due to the rising body mass index in these age groups [1]. The usual symptoms of SCFE include pain in the hip or knee and associated limping. An examination of the hip typically reveals limited internal rotation and flexion [2]. Early diagnosis and treatment are crucial to prevent complications and further damage. The primary aims of treatment are to prevent further slip of the epiphysis and reduce the resulting cam-deformity by repositioning or osteotomy. In situ fixation, such as screws through the physis, is a relevant treatment for stable and unstable slips with less than 30° of deformity and in prophylactic contralateral normal hips. Slips greater than 30° require anatomical realignment to restore hip anatomy and reduce the risk of avascular necrosis [3]. Although the primary aim of treatment is to minimize complications, the complication rate of SCFE remains high. Reported complications include avascular necrosis of the femoral head, chondrolysis, SCFE-induced impingement and associated articular cartilage damage and labral injury, fixation failure with deformity progression, growth arrest, and the development of bilateral disease [4].

Few studies have focused on gait kinematics following SCFE. In a study by Song et al. [5], gait outcomes in 30 patients with mild-to-moderate slips treated by in situ fixation were investigated. The study reported kinematic abnormalities that increased in proportion to the slip angle on the affected side, particularly when the slip angle was greater than 30°. They also observed some changes on the unaffected side. Westhoff et al. [6] recorded three-dimensional kinematic deviations in 39 patients who were skeletally mature and had mild SCFE. However, the patients had been treated with different methods, which could have decreased impingement and improved their gait. Sangeux et al. [2] reported data in adolescents with moderate-to-severe SCFE, managed by a single method, and included measurements of the extent of gait deviations. They found considerable gait abnormalities affecting both the affected and unaffected sides. Their findings indicated that the most significant deviations were observed in the transverse plane, including hip rotation and foot progression, as well as a delayed shift from adduction to abduction of the affected side when compared to a normal gait. These deviations were also linked to an upward tilt of the pelvis on the affected side.

Limited mobility in the lower limb joints can significantly alter the way pressure is distributed on the foot, especially when hip joint movement is restricted. Children who suffer from SCFE experience abnormal movement patterns, decreased loading on the affected leg, and often have severe walking disabilities. 

To understand how force from the ground is received by the foot and how the foot contacts the ground, plantar pressure should be assessed [6]. Plantar pressure measurement during bipedal standing provides important data of foot loading under different postural activities [7]. The capacitive sensors are common pressure sensors used for the assessment of plantar pressure [8]. 

If we can identify a relationship between these disabilities and plantar pressure distribution, then evaluating plantar pressure distribution could become clinically significant [9].

The rationale behind conducting the present study was to consider children with unilateral SCFE who underwent surgical treatment by in situ fixation with a single percutaneous screw. This group of patients is not accurately assessed in terms of plantar pressure compared to children with other types of lower limb fractures. The hypothesis of this study was that there would be differences in the static plantar pressure parameters between the affected and unaffected limb. The objective of our study was to analyze the static plantar pressure in children with surgically treated unilateral SCFE under different conditions (eyes open, eyes closed, and head retroflexed).

## 2. Materials and Methods

### 2.1. Participants 

This study was conducted between October 2023 and December 2023. The selection of children was made from the patients referred to the Pediatric Surgery department of “Louis Turcanu” Emergency Children’s Hospital in Timisoara, Romania. The inclusion criteria were children with unilateral SCFE of the dominant leg who were treated by surgery (in situ fixation with one percutaneous screw), at least 3 months after surgery. The dominant leg was determined as the leg the participant self-reported as their preferred leg for kicking [10].

Exclusion criteria included additional injuries apart from SCFE, secondary avascular necrosis of the femoral head, secondary chondrolysis of the hip, other musculoskeletal disorders, a history of neurological diseases, vestibular or visual disturbances, or any other pathology that would impair their motor performance. Children younger than 5 years old were also excluded. One child was excluded due to cerebral palsy. Therefore, 22 patients were enrolled in the study, which is the minimum sample size of 20 subjects calculated using G*Power (Heinrich-Heine-Universität, Düsseldorf, Germany) with a significance level of 0.05, 0.95 power, and an effect size of 0.8 [11].

The patients’ characteristics that were collected include age, height, weight, body mass index, and leg length, which was measured in centimeters with children lying supine from the anterior superior iliac spine to the ipsilateral medial malleolus with a standard tape measure on each lower limb (Table 1). Leg length discrepancy (LLD) was also considered. The range of motion of both hips was recorded (flexion, extension, abduction, adduction, and internal and external rotation assessed with the hip in 90° of flexion) (Table 2). The hip range of motion was measured by goniometry using a conventional manual goniometer. This method has high reliability when all the six arcs of hip motion are summed up [12] and is the first-choice tool for the assessment of hip range of motion in clinical settings [13]. 

All participants were right-leg-dominant.

Participation in this study was completely voluntary and all participants’ parents provided written informed consent. This study was conducted in accordance with the Declaration of Helsinki and was approved by the Ethics Committee of the “Victor Babes” University of Medicine and Pharmacy in Timisoara (reference no. 46/02.10.2023).

### 2.2. Assessment 

The Plantar pressure analysis PoData system (made by Chinesport, Udine, Italy) was used to assess plantar pressure. This system provides information about weight distribution on the right and left foot [14,15]. 

**Figure 1:**
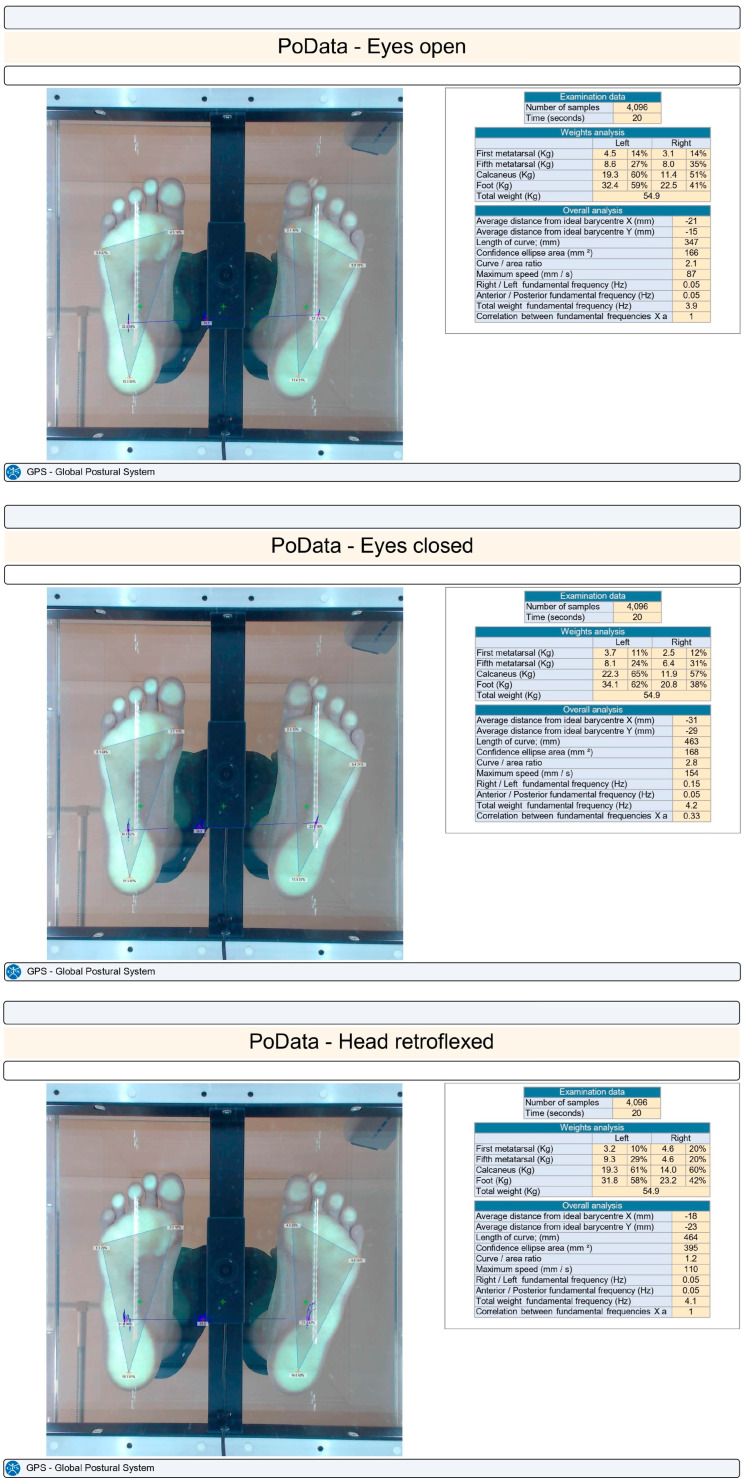
Plantar pressure results in the three testing conditions (eyes open, eyes closed, head retroflexed).

Participants were asked to stand on the platform barefoot with their lower limbs extended, arms positioned naturally along their sides, eyes opened, and in an upright posture for 20 s (Figure 1). They were instructed to keep their natural feet position, look ahead, and fix their gaze on a target point on the wall without talking or moving. The test was considered invalid if the child moved or lifted an arm, lifted their forefoot or heel, fell out of position, moved their head, or talked. Plantar pressure was recorded on three anatomical regions: first and fifth metatarsal heads and heel, on both the right and left foot [16].

This study calculated the percentage of body weight distribution in different areas. A perfect load for an ideal subject has the following distribution: 1/6 (16.67%) of total weight on the fifth metatarsal head, 2/6 (33.33%) of total weight on the first metatarsal head, and 3/6 (50%) of total weight on the heel [17].

The measurements of the static plantar pressure were performed for each subject under the following conditions: with their eyes open, with their eyes closed, and with their head retroflexed. The subjects did not change foot positions between the testing conditions. The measurement was performed once for each condition (eyes open, eyes closed, and head retroflexed). 

### 2.3. Statistics

All statistical analyses were performed using GraphPad Prism 5.0 for Windows. Descriptive statistics were computed for all variables (mean and standard deviation). Before statistical applications, the normal distribution of values in this study was verified by the D’Agostino–Pearson normality test. Comparisons between the plantar pressure distribution of the right and left lower limbs were performed by Student’s unpaired *t*-test or a chi-squared test. Paired *t*-tests were used to compare the intragroup data (plantar pressure distribution of the same lower limb in the three different conditions). A *p*-value less than 0.05 was considered statistically significant [18].

## 3. Results

The time between surgery and assessment ranged between 5 months and 4 years. Fourteen of the twenty-two patients (63.6%) were between 5 months and 13 months after surgery. The other eight children had 36 to 48 months between intervention and plantar pressure evaluation. The median follow-up was 20.7 ± 17.5 months.

The total foot loading was significantly higher on the unaffected limb compared with the affected limb (*p* < 0.001) for all three testing conditions (eyes open, eyes closed, and head retroflexed). The loadings on the first metatarsal were significantly increased on the right foot (affected limb) when compared with the left foot (unaffected limb) in all testing conditions (*p* < 0.05). The loading on the fifth metatarsal was significantly increased on the right foot when compared with the left foot in the eyes-open condition. In contrast, when assessed with the eyes-closed and head-retroflexed conditions, there were no differences between the right and left loading on the fifth metatarsal. The heel loading was significantly smaller on the right foot (affected limb) in comparison to the left one (unaffected limb) in all three testing conditions (*p* < 0.05). The results of the plantar pressure analysis are presented in Table 3.

When assessing the differences between the testing conditions, there were no significant differences for the right and left foot loadings, or for the three sites of weight distribution, except for the right fifth metatarsal head (lower loading in eyes-closed condition in comparison to eyes open; *p* = 0.0068), left fifth metatarsal head (increased loading in head-retroflexed condition in comparison to eyes open; *p* = 0.0209), and left heel (lower loading in head-retroflexed condition in comparison to eyes open; *p* = 0.0293). 

In 14 of the 22 patients (63.6%), the right lower limb (affected side) was shorter than the left one (unaffected side). The other eight children had no leg length discrepancy (LLD). Since the left healthy limb was longer or equal to the affected part, we decided to investigate a possible correlation between the LLD and the plantar pressure parameters of the left foot. The correlations were recorded for all three testing conditions (Table 4). We found significant positive correlations between LLD and left fifth metatarsal head loadings in the eyes-open, eyes-closed, and head-retroflexed conditions. In contrast, a significant indirect correlation was recorded between LLD and left heel loading. 

However, no significant correlations were found between LLD and the left foot loading in any of the testing conditions (eyes open, eyes closed, and head retroflexed). 

## 4. Discussion

The current study aimed to investigate the static plantar pressure in children who have had surgery for unilateral SCFE. Our study is distinctive because it investigates the distribution of static plantar pressure on the first and fifth metatarsal heads and the calcaneus of both the right and left foot in various conditions. These conditions include having the eyes open, having the eyes closed, and having the head retroflexed. 

All our patients had a follow-up less than or equal to 48 months. According to Ahmad et al., a minimum mean orthopedic follow-up of a short-term study should be 30 months [19]. In our study, 6 of the 22 participants (27.2%) had at least 30 months of follow-up. Due to the timing of surgical intervention, a comparison between patients with short-term follow-up and those with mid-term follow-up (at least 60 months) could not be performed.

When compared to the healthy hip joint, the patients had a significantly reduced range of motion of the affected hip for all movements, except for abduction. The findings of Bartolo et al.’s [20] study suggest that when the hip joint is restricted during the gait, there is a significant increase in peak plantar pressures and pressure-time integrals (PTI) in the first metatarsal region of interest. This indicates that the first metatarsal may experience higher stress due to a reduced hip joint range of motion during the midstance and/or toe-off stage of the gait. Additionally, when the hip joint restriction is induced, the contralateral heel region exhibits higher PTIs, suggesting that weight is shifted abruptly to the opposite limb during the gait, particularly during heel strike. Their study found that PTI in the heel region was significantly increased due to hip joint restriction.

Children who have experienced SCFE often develop a discrepancy in leg length. This is made worse by the premature closure of the growth plate due to the in situ fixation of the slipped epiphysis. Since SCFE usually occurs before the skeletal maturity of the child, the difference in leg length can worsen over time. This inequality in leg length is often linked to abnormal walking patterns, which can cause arthritis in the lower extremities and lumbar spine during adulthood [21]. In our study, the LLD ranged between 0.2 and 1.5 cm; the affected limb was shorter than the healthy one. In eight patients, there was no LLD. According to a study by Kim SJ et al. [21], the average discrepancy in leg length (LLD) was 1.4 cm six years post-surgery. The study was conducted on 85 patients to determine the correlation between the degree of slip and the magnitude of LLD. They [21] found that patients with a high degree of slip were more likely to develop clinically significant LLD.

In a survey conducted by Gross et al. [22], 74 patients who had an LLD of 1.5 cm or more were studied. The findings showed that patients with a difference of less than 2.0 cm did not experience any issues with their shorter leg. As the difference increased, there were more problems, but there was no specific “cutoff” point. Therefore, it can be concluded that there is little reason to equalize a discrepancy less than 2 cm.

Gait analyses have indicated that if there is a difference in leg length of more than 1 cm, it can lead to gait asymmetry. As the difference in leg length increases, the asymmetry also increases and limping becomes noticeable [23]. For patients with a moderate LLD of 2 cm to 5 cm, conservative treatment is usually recommended. Experts generally agree that LLD should be corrected up to 1 cm for growing children and 2 cm for adults.

Insoles and shoe lifts are two ways to equalize leg length. However, the shoe’s volume can limit the effectiveness of insoles. Heel wedge insoles can correct an LLD of up to 2 cm, while sole lifts can correct up to 5 cm with closed shoes.

Orthosis is another option for an LLD exceeding 5 cm. However, shoe lifts of 5 cm or more can lead to increasing instability, making the use of an orthosis necessary.

Surgical treatment is an alternative option for patients with an LLD of 2 cm or more [23]. After surgery, patients continue to have an uneven distribution of weight on their feet, with the affected limb bearing significantly less weight for a period of time. These differences were recorded in all three testing conditions. When referring to the affected limb tested with eyes open, we noticed increased weight loads on the first and fifth metatarsal heads with a remarkably decreased weight load on the heel in comparison to the unaffected part. These differences were also recorded when tested with eyes closed and with the head retroflexed, except for the fifth metatarsal head loading (no significant differences between the affected and unaffected limb). 

The study of De Blasiis et al. [24] assessed the plantar pressure parameters under open-eye and closed-eye conditions in 20 healthy young subjects (mean age 20.2 ± 0.9 years). Two visual conditions revealed significant differences between the left and right sides, with the right side showing larger parameters under both visual conditions, except for the rearfoot. 

In our study on 22 children after a surgically treated condition affecting the hip joint, there were significant differences between the right and left sides in both open-eye and closed-eye conditions, except for the fifth metatarsal head (no significant difference when assessed with eyes closed).

Nisand et al. [25] investigated the effects of backward head movement on plantar pressure. The study involved testing healthy young adults (with an average age of 20.6 ± 1.3 years) in two positions: one where the head was in a natural position (with varying head-board distance among participants) and the other where the head was no longer in its natural position, but in contact with the backboard. The results of the study revealed that aligning the head over the trunk led to an increase in forefoot plantar pressure. In our research, when referring to the unaffected limb, we noticed an increased loading on the fifth metatarsal head and a lower calcaneus loading when assessed with the head retroflexed in comparison to the natural eyes-open condition. 

Considering LLD, we performed correlation tests between this variable and static plantar pressure parameters of the healthy unaffected lower limb. Miura et al., assessing the plantar pressure distribution during standing in women with end-stage hip osteoarthritis, observed that as LLD increased, the heel was less frequently observed to be the maximum plantar pressure region [9]. In our study, we recorded that as the LLD increased, the heel loading of the unaffected side decreased in all three testing conditions. However, this correlation is significant only when assessed with eyes closed.

In contrast to previous studies, we focused on pediatric patients with unilateral SCFE who were treated with in situ fixation using a single percutaneous screw, which is the standard treatment for SCFE in many studies [26,27,28]. 

In our practice, we used intra-operative fluoroscopic imaging for guiding the placement of the screw guide pin. After the confirmation of the drill trajectory, the final screw was placed. The screw needs to be precisely positioned perpendicular on the physis and deep and central in the femoral head. Inappropriate screw placement can result in significant complications such as penetration of the hip joint, chondrolysis, vascular injury of the femoral head, avascular necrosis, and poor patient results [29].

The goals of screw fixation for the treatment of SCFE are stabilizing the physis, preventing further slippage, avoiding avascular necrosis or chondrolysis, and promoting physeal closure. A technique that utilizes a single hip screw has shown to provide a good outcome in the short and intermediate term [26,30]. A study by Jamil et al. [30] showed that screw positioning other than in the center of the femoral head can also provide physeal stability and has no correlation with the timing of epiphyseal closure and the risks of avascular necrosis and chondrolysis in mild cases. Attempting to achieve the central-central position may increase the potential hazard of joint surface penetration and damage to capsular vessels; therefore, the risk of chondrolysis and avascular necrosis increases further.

Although secondary avascular necrosis of the femoral head and secondary chondrolysis of the hip are complications following SCFE, none of our patients presented these conditions when assessed for inclusion in this study. Disability due to the primary condition can be serious if not early diagnosed and properly treated. The prediction of disability related to a musculoskeletal condition is envisaged when assessing the long-term outcomes [31].

Our patients did not develop any complications following SCFE. However, the kinematics of the lower limbs were altered, as there persists an imbalance between the unaffected limb and the affected limb that underwent a successful surgical procedure. 

The maintenance of a stable balance during standing activities is important for fall prevention. The activities performed with eyes open (for example, throwing or catching a ball in a stable standing position) are frequent in an active population group (children with an age range between 11 and 17 years). Our patients, even after surgery for a musculoskeletal disorder, should be encouraged to get engaged in sport and recreational activities. Children have to take part in age-specific activities. Adequate growth and development, without the fear of overloading the affected, surgically treated hip should be considered an objective for the overall management of children diagnosed with SCFE. 

The decrease of stability in the standing position with eyes closed or with the head retroflexed (for example, when participating in games and creative exercises or when engaging in video games) were the challenges when we chose to investigate the plantar pressure in different conditions. The involvement of an active age group in recreative activities in safety conditions should be the starting point of postural balance retraining. After analyzing the plantar pressure results, the orthopedic surgeon who treated our patients recommended physical therapy for stability and adapted physical fitness.

The current research has limitations due to the absence of gait analysis and dynamic plantar pressure assessment. However, despite the small number of patients enrolled in our study, we were able to avoid biases and obtain a more accurate analysis of the plantar pressure by implementing selective inclusion and exclusion criteria. The short-term follow-up of most participants can be regarded as a limitation of the present study.

We aim to continue our research by including a group of children with surgically treated unilateral SCFE who will follow a supervised rehabilitation program. The analysis of the static plantar parameters in two different groups (with and without a supervised rehabilitation program) will provide more data regarding the functional outcomes of children with SCFE treated by in situ fixation with one percutaneous screw.

## 5. Conclusions

The static plantar pressure in surgically treated unilateral SCFE showed differences between the affected and unaffected limbs, even though it was assessed at least 5 months after the intervention. This category of pediatric patients, even after a successful surgical procedure, presented asymmetric foot loadings that can impact postural static activities in different conditions (natural eyes-open, eyes-closed, or head-retroflexed posture).

## Figures and Tables

**Table 1:** Patients’ characteristics.

Variables	Patients (N = 22)
Age (years) *	14 ± 2.8 (11–18)
Gender	
Male, N	20
Female, N	2
Height (cm) *	166.4 ± 9.1 (152–182)
Weight (kg) *	69.42 ± 13.22 (55–95)
BMI (kg/m^2^) *	25.09 ± 4.2 (18.23–32.87)
Leg length discrepancy (cm) *	0.5 ± 0.051 (0–1.5)

* Data are presented as mean ± standard deviation (range); N: number of patients; BMI: body mass index.

**Table 2:** Range of hip motion.

Motion	Affected Limb Mean ± SD *	Unaffected Limb Mean ± SD *	*p*
Flexion (°)	84.5 ± 8.2 (75–100)	91.3 ± 11.9 (80–120)	**0.003**
Extension (°)	21.8 ± 2.4 (20–25)	24 ± 3.6 (20–30)	**0.02**
Abduction (°)	40 ± 13.8 (15–60)	45.4 ± 12.6 (25–70)	0.17
Adduction (°)	17.7 ± 5.9 (10–30)	20.9 ± 4.2 (15–30)	**0.047**
Internal rotation in 90° of flexion (°)	25 ± 21.9 (0–75)	38.6 ± 21 (30–85)	**0.019**
External rotation in 90° of flexion (°)	36.3 ± 19.2 (15–60)	49.5 ± 19.2 (30–90)	**0.028**

* Data are presented as mean ± standard deviation (range); SD: standard deviation. The bolded *p*-values indicate significant differences.

**Table 3:** Pressure load distribution (percentage of body weight on each foot) in testing conditions.

Variables	Eyes Open	*p* ^#^	Eyes Closed	*p* ^#^	Head Retroflexed	*p* ^#^
**Right foot load** (%)	42.82 ± 5.58	**<0.0001**	43.55 ± 7.04	**<0.0001**	43.09 ± 7.04	**<0.0001**
**Left foot load** (%)	57.18 ± 5.58		56.45 ± 7.04		56.91 ± 7.04	
**Right MT1** (%)	24.18 ± 8.41	**0.001**	25.18 ± 6.74	**<0.0001**	26.09 ± 8.15	**0.003**
**Left MT1** (%)	16.27 ± 6.4		16.18 ± 7.08		18.09 ± 9.03	
**Right MT5** (%)	38.91 ± 11.97	**0.005**	37.27 ± 11.13	0.083	38.36 ± 12.9	0.08
**Left MT5** (%)	30.27 ± 7.05		31.64 ± 10.32		32.18 ± 9.81	
**Right heel** (%)	35.55 ± 10.51	**<0.0001**	37.27 ± 11.13	**<0.0001**	35.73 ± 11.3	**0.001**
**Left heel** (%)	53.82 ± 9.34		52.36 ± 10.71		49.82 ± 14.81	

MT1: first metatarsal head; MT5: fifth metatarsal head; ^#^ comparison between the right and left limb (unpaired *t*-test). The bolded *p*-values indicate significant differences.

**Table 4:** Correlation of left plantar pressure parameters with leg length discrepancy.

Plantar Pressure Parameters	Leg Length Discrepancy	
	r	*p*
Eyes open		
Left foot load	0.254	0.25
Left MT1	−0.296	0.18
Left MT5	0.713	**0.0002**
Left heel	−0.326	0.13
Eyes closed		
Left foot load	0.265	0.23
Left MT1	−0.334	0.12
Left MT5	0.665	**0.0007**
Left heel	−0.425	**0.048**
Head retroflexed		
Left foot load	−0.087	0.69
Left MT1	−0.101	0.65
Left MT5	0.699	**0.0003**
Left heel	−0.395	0.068

r: rank correlation coefficient; *p*: *p*-value. The bolded *p*-values indicate significant differences.

## Data Availability

The data presented in this study are available on request from the corresponding author (A.D.B.) due to privacy.
